# A Pilot Study of Parent Mentors for Early Childhood Obesity

**DOI:** 10.1155/2016/2609504

**Published:** 2016-06-09

**Authors:** Byron A. Foster, Christian A. Aquino, Mario Gil, Jonathan A. L. Gelfond, Daniel E. Hale

**Affiliations:** ^1^Division of Inpatient Pediatrics, Department of Pediatrics, University of Texas Health Science Center at San Antonio, 7703 Floyd Curl Drive, MC 7803, San Antonio, TX 78229, USA; ^2^Regional Academic Health Center Clinical Research Unit, University of Texas Rio Grande Valley, 2102 Treasure Hills Boulevard, Harlingen, TX 78550, USA; ^3^Department of Epidemiology & Biostatistics (DEB), University of Texas Health Science Center at San Antonio, 7703 Floyd Curl Drive, MC 7933, San Antonio, TX 78229, USA; ^4^Division of Endocrinology, Department of Pediatrics, University of Texas Health Science Center at San Antonio, 7703 Floyd Curl Drive, MC 7806, San Antonio, TX 78229, USA

## Abstract

*Objective.* To assess the feasibility of a parent mentor model of intervention for early childhood obesity using positive deviance-based methods to inform the intervention.* Methods.* In this pilot, randomized clinical trial, parent-child dyads (age: 2–5) with children whose body mass index (BMI) was ≥95th percentile were randomized to parent mentor intervention or community health worker comparison. The child's height and weight were measured at baseline, after the six-month intervention, and six months after the intervention. Feasibility outcomes were recruitment, participation, and retention. The primary clinical outcome was BMI *z*-score change.* Results.* Sixty participants were enrolled, and forty-eight completed the six-month intervention. At baseline, the BMI *z*-score in the parent mentor group was 2.63 (SD = 0.65) and in the community health worker group it was 2.61 (SD = 0.89). For change in BMI *z*-score over time, there was no difference by randomization group at the end of the intervention: −0.02 (95% CI: −0.26, 0.22). At the end of the intervention, the BMI *z*-score for the parent mentor group was 2.48 (SD = 0.58) and for the community health worker group it was 2.45 (SD = 0.91), both reduced from baseline, *p* < 0.001.* Conclusion.* The model of a parent mentor clinical trial is feasible, and both randomized groups experienced small, sustained effects on adiposity in an obese, Hispanic population.

## 1. Introduction

The prevalence of obesity in early childhood has shown some signs of decreasing; however, for the Hispanic population, it continues to remain high, estimated at 16.7% for 2–5-year-old Hispanic children in the United States, double that of the general population [1]. There are few effective interventions that have enrolled obese, Hispanic children from this age group [2]. There are also very few prevention studies shown to be effective in Hispanic families [3, 4]. Of the intensive, clinical interventions shown to be effective, a major limitation is the intensity required as this limits the intervention's potential public health scope and brings about challenges of sustainability [5–8].

Peer or parent mentors are an alternative to professional counseling for delivering information and assisting with behavioral change. Parent mentors have been used as an effective intervention model in coaching other parents on their child's diabetes management [9] and in childhood asthma [10]. This model may also be an attractive option for the treatment of early childhood obesity because it requires a relatively low amount of resources to initiate a parent mentoring program, and there is potential for empowerment and sustainability within the community. The feasibility of a parent mentor model for obesity has not been examined.

Head Start is a government-funded program for low-income families that includes early learning and school readiness, health and development screenings as well as daily meals, and family well-being to strengthen parent-child relationships. One of Head Start's core values is the empowerment of families, and they serve a population at high risk for obesity; therefore, the parent mentor model of intervention meshes well with their structure and operational values. Head Start programs offer services to nearly one million students [11]. The data on obesity interventions in Head Start centers in other locations have yielded variable results.

The positive deviance approach was used as the premise for building the intervention as it is well suited for high-risk populations and family empowerment. Positive deviance is the idea that, even among those most at risk for an adverse outcome, there are some individuals in the community who find a way to succeed, and, by identifying how those individuals or families succeed, one can identify successful strategies and behaviors that utilize local resources and knowledge [12]. This approach was historically used in malnutrition [13] though it has recently been explored in obesity [14–17].

This study was designed to evaluate the feasibility of a clinical trial to test the hypothesis that parent mentors using positive deviance-derived education could be an effective intervention for early childhood obesity in the context of a Head Start program serving a low-income, Hispanic community.

## 2. Methods

### 2.1. Study Design

This was a pilot, randomized clinical trial designed to evaluate the feasibility of an intervention using parent mentors trained in positive deviance behaviors to reduce adiposity among obese 2–5-year-old children. We used community health workers providing education as our comparison group. All of the parent-child dyads recruited for the study were actively enrolled in a Head Start program, Neighbors in Need of Services (NINOS Inc.). The full protocol for this trial is described in greater detail elsewhere [18] and is also available by contacting the corresponding author.

### 2.2. Sample Selection and Recruitment

NINOS Inc. staff identified 139 families with at least one 2–5-year-old obese child from the 16 centers in the geographic area of this study, Cameron County, South Texas. Identified families were contacted by letter and phone to explain the study and determine interest in participating. Eligibility was determined as having a body mass index (BMI) ≥95th percentile for age and gender. Exclusion criteria were intellectual disability, severe development delay, seizure disorder, diabetes, cerebral palsy, any genetic problem, and inability to communicate in either English or Spanish. Sixty parents provided written consent, and they and their child were enrolled in the study. Parent-child dyads were randomized 1 : 1 to intervention or comparison in blocks of six using REDCap [19]. The randomization allocation table was generated by a research assistant and was concealed from the principal investigator and staff. Enrollment and assignment of participants to intervention and comparison arms were carried out by clinical research unit staff. The staff were not blinded to assignment after enrollment. Recruitment occurred between January and February 2015 (4-week period).

### 2.3. Parent Mentor Recruitment and Training

Parent mentors were recruited from Cameron County Head Start centers and trained as described previously [18]. Briefly, these individuals were required to have a 2–5-year-old child at a healthy weight who was enrolled in Head Start at the time; the parents themselves could be of any weight. Their education varied from some high school to some college. They were selected by the Head Start staff for their leadership qualities. Four parents received a one-day intensive training on the content of the parent mentor manual and on reflective listening, with three parents selected for participation based on their engagement in the training. The parent mentor manual was developed using the American Academy of Pediatrics guidelines on obesity prevention [20] and the previous study on identified positive deviance practices done in Cameron County [16]. There were five main foci that the parent mentors were instructed on as being potentially effective strategies to share with their parent mentees: dealing with behavior problems without using food, identifying internal motivators for healthy habits, organizational strategies for feeding, accurate perceptions of weight, and effective snacking strategies. They completed worksheets monthly for each encounter with their mentee and reported what topics were discussed. They were compensated for their time with $50 per parent per month.

### 2.4. Intervention

Parent-child dyads randomized to the intervention arm were assigned to one of three parent mentors. Home assessments with the parent and child were conducted by each parent mentor at baseline and three months after enrollment. A standardized approach to the visit included asking about five main areas (positive deviance behaviors listed above), and then the parent mentors provided coaching on those areas. At least one phone call per month was made by each parent mentor in order to reinforce those behaviors. Intervention parents also participated in community meetings held at Head Start centers. Meetings were conducted on a monthly basis by parent mentors, and each mentor was allowed to implement her own curriculum in accordance with the goals discussed with the participating group. The community meetings for intervention and comparison participants were separately scheduled and located.

#### 2.4.1. Comparison Condition

Parent-child dyads randomized to the community health worker comparison arm had the opportunity to attend one of three monthly community meetings held at Head Start centers. These meetings were conducted by a local* “promotora”* or community health care worker. In contrast to the intervention arm meetings, the comparison arm meetings followed a structured setting outlined by the EatPlayGrow*™* Curriculum [21]. Topics discussed during the meetings included health benefits of fruits and vegetables, limiting unhealthy foods, physical activity, portion control, and sleep, using an interactive format via songs, exercise, story time, and snacks. During the hour-long meeting, the first ten minutes were reserved for signing in and serving fruits and vegetables, the following forty-five minutes were for content delivery, and the final five minutes were used to remind parents of the following meeting and discuss any questions. Every parent kept a journal to log thoughts and perceptions about each meeting. Homework handouts were administered to the parents and children. Children were allowed to attend the meetings with their parents but it was not a requirement. In contrast to the intervention arm, parent-child dyads did not receive any home visits or follow-up phone calls throughout the study. The only contact time between the community health worker and parents was during the hour-long community meeting once a month.

#### 2.4.2. Control Group

An interim analysis showed no difference between the two comparison arms of the study. Therefore, a control group was identified with no intervention other than usual care in Head Start which consists of providing healthy meals and messaging on healthy habits in newsletters. Head Start staff weigh and measure each child at enrollment and every six months. The control group children were identified as obese (≥95th BMI percentile for age and gender), were enrolled in Head Start over the same period of time, attended the same local Head Start centers, and were matched to each study participant by sex and on their initial BMI *z*-score (within 0.3 units). The average baseline BMI *z*-score difference between participants in the trial and community control group children was 0.03 units.

#### 2.4.3. Outcome Measures

All measures described below were administered in either English or Spanish depending on the preference of the individual participant. The primary outcome was BMI *z*-score change at the end of the six-month intervention; BMI *z*-scores were calculated using Centers for Disease Control standards [22]. A postintervention follow-up was completed at 12 months from initial enrollment (six months after intervention). Children were measured and weighed without shoes and in light clothes by trained research staff. Secondary outcome measures assessed at baseline, at the end of intervention (six months), and six months after intervention (12 months from baseline) included health-related quality of life using the Pediatric Quality of Life Inventory (PedsQL 4.0, Mapi Research Trust, Lyon, France) [23], feeding behaviors measured by the Comprehensive Feeding Practices Questionnaire (CFPQ) [24], dietary intake assessed using the Block Kids Food Screener (BKFS) developed by NutritionQuest (Berkeley, CA, USA) [25], screen time, sleep, and outside play using standardized questions previously described [18, 26–28]. Growth (height) velocity was calculated over a twelve-month period, and each individual was plotted on a sex specific growth velocity chart [29].

#### 2.4.4. Feasibility Outcomes

We focused on recruitment, retention, and participation outcomes for our feasibility assessment. Recruitment was evaluated by assessing how many parent-child dyads were screened and contacted to achieve one dyad enrolled. Retention was assessed as the proportion of subjects completing the final study visit. Participation was calculated using a point system for each potential interaction in each arm of the study with fourteen possible points in the intervention arm and six points possible for the comparison arm. These points were categorized into high participation ≥ 65%, some participation ≥ 1% and < 65%, and no participation = 0%.

### 2.5. Statistical Analysis

The primary clinical outcome of BMI *z*-score was analyzed under the intention-to-treat principle. Linear mixed models with a random intercept were used to evaluate the primary outcome using randomization group, time (0, 6, and 12 months coded as intervals rather than a continuous variable to evaluate period effects), and their interaction (group × time) as main effects adjusted for baseline measurements; an interaction term between baseline measurement and time was included if significant to account for the intercept variation. This modeling approach was also used for the secondary outcomes of the PedsQL 4.0 scales, CFPQ scales, and diet and activity measures. Data were analyzed using SPSS (version 23; IBM SPSS Statistics, IBM Corporation, Chicago, IL).

#### 2.5.1. Sample Size and Power

Weight maintenance in this age group with continued growth in height leads to a decrease in BMI *z*-score among obese children of about 0.5 units over 6 months; this approximates a moderate effect size. With an expected mean BMI *z*-score of 2.5 at baseline, a standard deviation of 0.5, and a two-sided *α* of 0.05, 30 participants in each group provided 48% power to detect a difference of moderate effect size, 86% power to detect a large effect size (reduction in BMI *z*-score of 0.8), and 12% power to detect a small effect size (reduction in BMI *z*-score of at least 0.2).

### 2.6. Ethics

This study was approved by the University of Texas Health Science Center at San Antonio Institutional Review Board. Both parent mentors and parents of obese children provided written informed consent to participate. This trial is registered at clinicaltrials.gov under NCT02373670. All parents were provided with participation stipends as previously described [18].

## 3. Results

**Figure 1:**
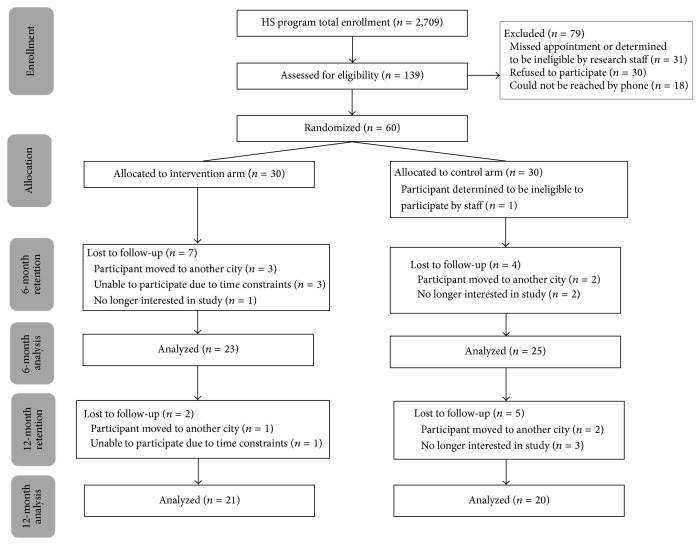
Recruitment and retention efforts.

The total program enrollment for the Cameron County Head Start includes more than 2,700 students. From this cohort of students, 139 families were assessed for eligibility to participate in the randomized clinical trial. Ultimately, 79 families were excluded and 60 were enrolled in the trial. Of the sixty parent-child dyads initially enrolled in the study, forty-eight completed the six-month follow-up visit (end of intervention) (80%) and forty-one completed the twelve-month visit (six months after intervention) (68.3%) (Figure 1). Participation in both the intervention and the comparison groups overall was high, with 76% of all participants meeting the benchmark of ≥65% of possible interactions to qualify as having high participation. Only two of the parent-child dyads had no participation recorded while still completing all study visits for measurements, both in the comparison arm of the study. In assessing adherence, the parent mentors followed a standardized approach of discussing five main areas (positive deviance behaviors) when conducting home visits and phone calls throughout the trial. Their discussion of each area by proportion of all documented interactions was perceptions of weight, 84.3% (95% CI: 75.9, 92.7); snacking strategies, 95.3% (95% CI: 91.6, 99.0); dealing with behavior problems and emotions, 69.2% (95% CI: 56.2, 82.2); organization and taking control, 77.1% (95% CI: 67.8, 86.5); and figuring out why healthy habits are important, 78.6% (95% CI: 69.3, 87.7).

Comparing the baseline demographics and anthropometrics, there were no significant differences between the randomized groups (Table 1). The parents enrolled were 100% Hispanic and had overall low income. There was no difference in baseline demographics between completers (*n* = 48) and noncompleters (*n* = 12) at six months. Those completing the twelve-month visit were less likely to be employed at baseline (44%) compared to noncompleters (78%), *p* = 0.05, and otherwise there were no differences.

For the outcome of BMI *z*-score, there was no difference in the mean change between the parent mentor and community health worker groups (mean difference: −0.02 (95% CI: −0.26, 0.22)). Both had a significant reduction in mean BMI *z*-score by time (*p* < 0.001, Table 2). Using estimated marginal means adjusted for baseline values, the estimated change in BMI *z*-score from baseline to end of intervention at six months was −0.24 (95% CI: −0.34, −0.15), and that between the end of intervention and the twelve-month time point was 0.00 (95% CI: −0.10, 0.11). When separated by baseline BMI *z*-score quartiles, participants starting at a higher *z*-score intercept had the largest overall *z*-score change with a mean of −0.68 (95% CI: −1.1, −0.24).

There was no effect on systolic blood pressure; diastolic blood pressure percentiles decreased overall by a mean of −5.53 (95% CI: −9.74, −1.31) at six months, with no effect by group (*p* = 0.96) (Table 2). For sleep, no differences by group were observed; the mean baseline for the parent mentor group was 10.7 hours (SD = 1.42) and was 10.7 (SD = 1.40) for the community health worker group. In the analysis adjusting for baseline and multiple measures, there was a significant increase from baseline to six months (*p* = 0.04) but then there was a decrease from six months to twelve months (*p* = 0.002) resulting in overall no difference between baseline and twelve months (*p* = 0.16) (Table 2). Weekday screen time had a trend toward significance in the interaction of randomization group × time (*p* = 0.08) with the intervention group decreasing screen time from a mean of 3.3 (95% CI: 2.3, 4.2) at six months to 2.1 (95% CI: 1.5, 2.7) at twelve-month follow-up.

Out of the forty-one participants who completed the six-month postintervention, 12.2% (5) had a growth (height) velocity < 50th percentile, 29.3% (7) were between the 50th and 90th percentile, and 70.7% (29) had a growth velocity > 90th percentile.

For the nonrandomized control group comparison, their baseline BMI *z*-score was 2.65 (95% CI: 2.38, 2.93) (Table 3) and their mean age at baseline was 45.6 months (95% CI: 44.0, 47.2). The difference between their baseline and six-month BMI *z*-score was not significant (*p* = 0.08, paired *t*-test). As a group over a twelve-month period, they did have a significant decrease in BMI *z*-score of −0.32 (95% CI: −0.53, −0.10), with a mean *z*-score at twelve months of 2.33 (95% CI: 2.02, 2.66).

Using the Block FFQ as the primary assessment of dietary intake, there was a significant reduction in sugary beverage intake overall with a mean change of −0.14 servings (95% CI: −0.23, −0.04) at six months but not by group (*p* = 0.96), with the significance occurring between baseline and end of intervention at six months (*p* = 0.001) and sustained with no change at twelve months (six months after intervention) (*p* = 0.83 for six versus twelve months) (Table 4). There was also a significant decrease in sugar added to food or drink over time of −1.22 tsp (95% CI: −2.12, −0.32), and no changes were seen by time or group for vegetable intake (*p* = 0.36 for time) or whole grain intake (*p* = 0.12). The overall caloric intake decreased between baseline and six months with a mean difference of −119.99 (95% CI: −252.23, 12.25 *p* = 0.07) and was then stable between end of intervention and twelve months (*p* = 0.51).

The quality of life scales assessed using the PedsQL 4.0 showed no significant changes from baseline to end of intervention or six months after intervention in either group, except in emotional functioning, with a significant increase occurring between baseline, 81.3 (95% CI: 76.9, 85.8), and end of intervention, 86.8 (95% CI: 83.1, 90.6) (*p* = 0.003). The following are mean overall scores for the intervention and comparison group throughout the trial: baseline, 84.4 (95% CI: 81.3, 87.5); end of intervention, 83.6 (95% CI: 79.2, 88.1); and six months after intervention, 85.7 (95% CI: 81.5, 90.0).

Finally, all of the CFPQ scales showed significance by time except the monitoring measure (*p* = 0.24) (Table 5). The “encourage balance and variety in diet” scale was the only measure to show significance by group (*p* = 0.009) with a higher score reported at the end of intervention and six months after intervention for the intervention group versus the comparison group, with mean score of 4.5 (95% CI: 4.2, 4.8), 4.8 (95% CI: 4.6, 5.0) for the intervention arm and 4.3 (95% CI: 4.0, 4.5), 4.5 (95% CI: 4.2, 4.7) for the comparison arm, respectively, over time. The scales that are most important and related to the intervention are environment, emotion regulation, restriction for health, restriction for weight control, and modeling. These scales were more heavily discussed during the parent mentor training before the beginning of the study.

## 4. Discussion

This is the first clinical trial to our knowledge that uses parent mentors as an intervention mechanism for childhood obesity. An important aspect of this trial was that it was conducted in a low-income, Hispanic population who, in the United States, are at higher risk of obesity compared with white children [1]. The data on recruitment, participation, and retention suggest that a full-scale clinical trial would be feasible in this setting. The high participation rates for both intervention and comparison groups in the present study indicate that our approach was successful in terms of engaging and motivating parents of obese children. Moreover, our findings provide evidence that parent mentors are effective at facilitating this type of engagement in their peers. It has been suggested that interventions sponsored or supported by government agencies and/or big organizations (i.e., top-down approaches) are more sustainable compared to bottom-up approaches like interventions driven by community-based organizations [30]. Our parent mentor intervention was driven by an independent social network, which is different compared to standard bottom-up approaches that are dependent on formal institutional support. This is significant because it suggests that a positive deviance approach, with peer mentors serving as agents of change, may hold the key to improving sustainability. At the same time, the relative success of these mentors may be dependent on their own capacities, and the variation between mentors in efficacy will need further testing in full-scale trial.

Both the parent-child dyads randomized to receive a parent mentor and those randomized to receive health education from a community health worker experienced a decrease in their adiposity as measured by BMI *z*-score. The diet and activity changes that were measured were consistent with this decrease in adiposity, and the plateauing of those diet and activity changes between the end of the intervention at six months and the twelve-month follow-up is consistent with their weight stabilization. We did not have a control group that was randomized to receive no intervention. A recent study comparing childhood obesity interventions with and without a true control group suggests that careful interpretation of results in those without a true control should be considered due to possible regression to the mean and biases favoring resolution [31]. For the control group that we did employ, the primary influence on them was their enrollment in the Head Start program. The reduction in the control group's BMI *z*-scores over twelve months makes it impossible to attribute the reduction seen in both of our intervention groups to the intervention. However, the lack of a significant effect in the first six months for the control children in contrast to the stronger reduction in the first six months of the two intervention groups suggests there is some additional benefit to receiving the intervention that should be evaluated in a larger clinical trial.

Other trials demonstrating success in reducing adiposity in this age group have utilized multidisciplinary teams including a dietician, psychologist, or behavioral counselor and some physical activity coaching [7, 8]. One trial specifically tested a mentoring component in addition to multidisciplinary education and found a significant benefit of health coaching by a paraprofessional mentor compared with the educational intervention alone [6, 32]. In a multidisciplinary intervention study with a usual care control [8], a larger effect size on BMI *z*-score was seen (mean difference −0.77, 95% CI: −0.27, −1.26) than in our study; other studies using a degree of intensity more similar to that described in this study (using motivational interviewing [5] or health coaches [6]) have seen a similar, more modest effect.

One important aspect of this study was its conduct in partnership with a Head Start program. While the Head Start program staff did not provide any direct education for the parents involved in the study, they did encourage their involvement. The potential for a scalable and effective program for reducing obesity has significant potential impact given the scope of Head Start and the population the programs serve [11]. Prior studies targeting obesity in Head Start have been mixed with one study showing no reduction in adiposity and even increases in some groups [33], whereas others have found decreases associated with usual Head Start participation [34]. Another series of trials found no significant effect with a weight-focused intervention [35] but then after including a parent-focused component found significant decreases from baseline in both the intervention and the control groups, suggesting a possible role for usual Head Start participation.

Using the CFPQ, we observed an increase in parental report of more child self-control of their own eating behaviors, a decrease in regulation of child's emotional states through use of food, an increase in parent promotion of well-balanced food intake, an increase in availability of healthy foods in the home, a decrease in use of food as a reward for child behavior, an increase in encouragement of child's own involvement in meal planning and preparation, an increase in parent demonstration of healthy eating, a decrease in parent pressure of child eating more food at meals, an increase in parent control of child's food intake to limit less healthy foods and maintain child's weight, and an increase in techniques to encourage consumption of healthy foods. One challenge of using the positive deviance method to develop the intervention is that there were no specific behavioral mapping tools for the targeted behaviors. However, given that one area of the positive deviance-based intervention was on how to address behaviors without food, the CFPQ findings are encouraging that the intervention had a causal relationship with the behavior changes reported. Interestingly, the only difference between the intervention and comparison group was an increase in parent reported encouragement of a balanced and varied diet in the intervention group. Prior observational studies have shown a correlation between restrictive feeding practices and increased risk for overweight [36–38]; however, some studies have suggested a role for restriction at least in early childhood [39], and the role of restrictive practices in the context of an intervention for obesity where a parent is trying to change patterns is less clear and should be explored in future interventional studies. Finally, reporting bias is certainly a possible influence on the CFPQ results, particularly after intervention.

The change in the health-related quality of life measure of emotional functioning may be a result of the interventions or relating to the natural maturation process. Also, the initial scale ratings for quality of life are lower than previously reported means of healthy children [40] and similar to that of another study of obese preschoolers [41]. However, these could also be unrelated to the child's weight status and be more related to their socioeconomic status or another confounding variable.

The limitations of this study include the secondary outcome measures of screen time, activity and sleep being by parental report, though any recall bias would probably have affected both groups equally. The limitation of no true control that was randomized is discussed above, though this study does add to the literature on describing the potential effect of usual Head Start participation. The study was underpowered to detect a difference between groups for a small effect size on adiposity, which is what we observed. However, the focus of this study was on feasibility of a novel intervention and study design. Finally, we cannot separate the effect of the content delivery versus the health coaching or support by the* promotora *with this study design.

## 5. Conclusion

A significant challenge in addressing the childhood obesity epidemic, particularly the disparities seen in minority populations, requires scalable and culturally acceptable interventions. The data presented suggest that the method of using parent mentors with minimal training is a potentially feasible intervention with a small effect size that warrants further exploration. Positive deviance as a method of deriving solutions from a high-risk community also warrants further testing in clinical studies. Finally, the role of early education centers, particularly Head Start, in addressing early childhood obesity is promising from both this and other recent studies.

## Figures and Tables

**Table 1:** Baseline demographic characteristics of study children and parents^(1)^.

Characteristics	Parent mentor group	Community health worker group	*p* ^(2)^
Child age, months, mean (SD)	54.4 (7.8)	51.1 (6.9)	0.08
Child sex, % female (*n*)^‡^	36.7% (11)	50.0% (15)	0.44
Parental age, years, mean (SD)	30.7 (7.6)	31.5 (6.9)	0.66
Parent sex, % female (*n*)^‡^	93.3% (28)	96.7% (29)	1.00
Preferred language, % English (*n*)^‡^	56.7% (17)	56.7% (17)	1.00
Parent BMI, mean (SD)	31.4 (8.3)	33.0 (6.7)	0.49
Hispanic, % (*n*)	100% (30)	100% (30)	
Household size, median (IQR)^+^	5.0 (1.3)	4.5 (3.0)	0.18
Income, % (*n*)^‡^			0.78
Less than $10,000	30.0% (9)	30.0% (9)	
$10,000–$25,000	30.0% (9)	43.3% (13)	
$25,000–$50,000	10.0% (3)	6.7% (2)	
Greater than $50,000	3.3% (1)	0.0% (0)	
Do not know/not sure	26.7% (8)	20.0% (6)	
Employment, % (*n*)^‡^			0.48
Employed/self-employed	46.7% (14)	63.3% (19)	
Unemployed/unable to work/student	30.0% (9)	20.0% (6)	
Homemaker	23.3% (7)	16.7% (5)	
Education, % (*n*)^‡^			0.62
Less than high school education	43.3% (13)	46.7% (14)	
High school graduate/GED	13.3% (4)	20.0% (6)	
Some college or technical school/college graduate	43.3% (13)	33.3% (10)	

^(1)^ *n* = 30 in the parent mentor group and community health worker group at baseline. GED: General Education Development Test; BMI: body mass index.

^(2)^ *p* values are calculated by independent-samples *t*-test unless otherwise noted. + denotes use of independent-samples Mann-Whitney *U* test; ‡: Fisher's Exact Test.

**Table 2:** Primary and secondary clinical outcomes^(1)^.

Measure^(3)^	Parent mentor group	Community health worker group	*p* ^(2)^		
			Group	Time	Group × time
*Primary outcomes*					
Child BMI *z*-score^a,c^			0.94	<0.001	0.94
Baseline	2.63 (2.4, 2.9)	2.61 (2.3, 2.9)			
6 months	2.48 (2.2, 2.7)	2.45 (2.1, 2.8)			
12 months	2.48 (2.2, 2.7)	2.31 (2.0, 2.6)			
Child BMI, kg/m^2^ ^b,c^			0.96	0.002	0.82
Baseline	21.1 (20.0, 22.3)	20.6 (19.6, 21.6)			
6 months	21.5 (20.0, 23.0)	20.9 (19.6, 22.2)			
12 months	22.4 (20.7, 24.1)	21.1 (19.6, 22.5)			
Child weight, lbs.^a,b,c^			0.49	<0.001	0.90
Baseline	54.1 (48.5, 59.7)	49.8 (46.2, 53.3)			
6 months	61.2 (54.3, 68.2)	56.5 (51.4, 61.5)			
12 months	67.2 (59.3, 75.1)	60.5 (54.2, 66.7)			
*Secondary outcomes*					
Blood pressure, systolic percentile			0.68	0.79	0.28
Baseline	70.0 (63.3, 76.8)	77.9 (72.3, 83.5)			
6 months	73.6 (66.4, 80.8)	71.8 (64.5, 79.1)			
12 months	71.0 (63.9, 78.1)	76.1 (68.6, 83.6)			
Blood pressure, diastolic percentile^a,c^			0.97	<0.001	0.98
Baseline	83.5 (79.4, 87.6)	87.2 (84.3, 90.1)			
6 months	79.1 (73.5, 84.7)	79.8 (75.3, 84.3)			
12 months	76.4 (71.1, 81.7)	76.7 (70.1, 83.3)			
Screen time, hrs. per weekday^b^			0.49	0.04	0.08
Baseline	3.0 (2.5, 3.6)	2.3 (1.7, 2.8)			
6 months	3.3 (2.3, 4.2)	2.1 (1.4, 2.9)			
12 months	2.1 (1.5, 2.7)	2.2 (1.6, 2.8)			
Screen time, hrs. per weekend day^a^			0.41	0.003	0.39
Baseline	4.6 (3.6, 5.5)	3.0 (2.2, 3.7)			
6 months	2.9 (2.2, 3.6)	2.7 (1.7, 3.8)			
12 months	3.6 (2.7, 4.5)	2.9 (2.1, 3.6)			
Active play time, mins. per day^b^			0.78	0.03	0.78
Baseline	120.0 (87.5, 152.5)	118.7 (90.9, 146.4)			
6 months	132.4 (94.0, 170.7)	145.4 (104.1, 186.8)			
12 months	107.9 (74.6, 141.1)	108.0 (72.0, 144.0)			
Sleep, hrs. per day^a,b^			0.55	0.007	0.83
Baseline	10.7 (10.2, 11.2)	10.7 (10.1, 11.2)			
6 months	11.2 (10.5, 11.8)	10.9 (10.1, 11.6)			
12 months	10.4 (10.0, 10.8)	10.4 (9.8, 11.0)			

^(1)^Values are means (95% CIs); *n* = 30, 23, and 21 in the parent mentor group and 30, 25, and 20 in the community health worker group at baseline and at 6 and 12 months, respectively.

^(2)^Linear mixed-model analysis on the entire study population; *p* values are for the individual effects of group and time as well as their interaction.

^(3)a^0–6-month mean difference significance at a *p* value < 0.05; ^b^6–12-month mean difference significance at a *p* value < 0.05; ^c^0–12-month mean difference significance at a *p* value < 0.05.

**Table 3:** Head Start comparison control data^(1)^.

Measure	Control	*p* ^(2)^	*p* ^(3)^	*p* ^(4)^
Child BMI *z*-score		0.08	0.29	0.005
Baseline	2.65 (2.38, 2.93)			
6 months	2.39 (2.02, 2.77)			
12 months	2.33 (2.02, 2.66)			
Child BMI, kg/m^2^		0.69	0.31	0.40
Baseline	20.5 (19.7, 21.3)			
6 months	20.0 (18.9, 21.0)			
12 months	20.8 (19.5, 22.1)			
Child weight, lbs.		<0.001	<0.001	<0.001
Baseline	46.4 (43.3, 49.4)			
6 months	49.2 (45.1, 53.3)			
12 months	53.9 (49.3, 58.5)			

^(1)^Values are means (95% CIs); *n* = 34, 24, and 34 at baseline and at 6 and 12 months, respectively.

^(2)^ *p* value calculated by paired-samples *t*-test between baseline and 6 months.

^(3)^ *p* value calculated by paired-samples *t*-test between 6 months 12 months.

^(4)^ *p* value calculated by paired-samples *t*-test between baseline and 12 months.

**Table 4:** Block Kids Food Screener results^(1)^.

Measure^(3)^	Parent mentor group	Community health worker group	*p* ^(2)^		
			Group	Time	Group × time
Fruit/fruit juice, cups^c^			0.47	0.01	0.79
Baseline	1.4 (1.0, 1.7)	1.3 (1.0, 1.6)			
6 months	1.3 (0.9, 1.6)	1.2 (0.9, 1.5)			
12 months	1.1 (0.8, 1.4)	1.0 (0.8, 1.2)			
Vegetables, cups			0.52	0.36	0.90
Baseline	0.5 (0.3, 0.6)	0.6 (0.2, 0.9)			
6 months	0.4 (0.3, 0.6)	0.6 (0.2, 0.9)			
12 months	0.4 (0.2, 0.6)	0.4 (0.2, 0.5)			
Potatoes, including French fries, cups			0.50	0.15	0.89
Baseline	0.2 (0.1, 0.3)	0.2 (0.1, 0.4)			
6 months	0.2 (0.1, 0.2)	0.2 (0.1, 0.3)			
12 months	0.2 (0.1, 0.2)	0.1 (0.1, 0.2)			
Whole grains, ounces			0.58	0.12	0.53
Baseline	0.5 (0.3, 0.7)	0.3 (0.2, 0.4)			
6 months	0.5 (0.3, 0.6)	0.5 (0.3, 0.7)			
12 months	0.5 (0.3, 0.7)	0.3 (0.2, 0.5)			
Saturated fat, grams			0.79	0.13	0.44
Baseline	15.2 (11.2, 19.3)	14.4 (7.8, 20.9)			
6 months	13.8 (8.1, 19.5)	13.1 (7.9, 18.4)			
12 months	13.3 (6.9, 19.8)	10.9 (8.4, 13.4)			
Meat, poultry, and fish, ounces			0.85	0.51	0.59
Baseline	1.8 (1.3, 2.4)	2.1 (0.8, 3.4)			
6 months	2.1 (1.0, 3.3)	2.1 (1.0, 3.3)			
12 months	2.2 (0.9, 3.4)	1.5 (1.0, 2.0)			
Dairy products, cups			0.52	0.06	0.77
Baseline	1.8 (1.4, 2.2)	1.4 (1.0, 1.8)			
6 months	1.5 (1.1, 1.8)	1.4 (1.0, 1.8)			
12 months	1.4 (1.0, 1.8)	1.2 (1.0, 1.4)			
Legumes, cups			0.85	0.19	0.18
Baseline	0.2 (0.1, 0.2)	0.2 (0.1, 0.2)			
6 months	0.1 (0.1, 0.2)	0.2 (0.1, 0.2)			
12 months	0.1 (0.1, 0.2)	0.1 (0.0, 0.1)			
Sugar added to foods/drinks, tsp^a,c^			0.54	<0.001	0.48
Baseline	5.9 (4.0, 7.7)	5.3 (3.6, 7.1)			
6 months	4.5 (2.2, 6.9)	4.3 (2.8, 5.8)			
12 months	4.7 (3.1, 6.2)	3.5 (2.4, 4.6)			
Sugars occurring in foods and juice, grams^a,c^			0.65	<0.001	0.53
Baseline	74.5 (58.2, 90.7)	68.4 (51.5, 85.2)			
6 months	62.5 (45.2, 79.8)	60.7 (44.1, 77.3)			
12 months	59.4 (43.6, 75.2)	48.7 (40.1, 57.3)			
Fiber, grams			0.43	0.06	0.83
Baseline	10.6 (8.1, 13.1)	11.2 (7.0, 15.4)			
6 months	9.6 (7.2, 11.9)	10.1 (6.0, 14.2)			
12 months	9.4 (6.4, 12.5)	7.5 (5.8, 9.1)			
Yogurt, containers per week			0.45	0.24	0.43
Baseline	2.9 (1.7, 4.0)	2.2 (0.9, 3.5)			
6 months	2.7 (1.4, 4.1)	2.3 (1.4, 3.3)			
12 months	2.7 (1.3, 4.2)	3.5 (1.6, 5.3)			
Sugary beverages, servings^a,c^			0.96	0.001	0.74
Baseline	0.3 (0.2, 0.4)	0.3 (0.2, 0.4)			
6 months	0.2 (0.1, 0.3)	0.2 (0.1, 0.2)			
12 months	0.2 (0.1, 0.2)	0.1 (0.1, 0.2)			
Energy from sugary beverages, kcals^a,c^			0.94	<0.001	0.63
Baseline	37.1 (14.7, 59.5)	40.2 (20.6, 59.8)			
6 months	18.2 (6.6, 29.7)	23.3 (12.0, 34.5)			
12 months	19.6 (9.0, 30.2)	17.7 (6.5, 28.9)			
Protein intake, kcal percentage			0.97	0.37	0.74
Baseline	17.1 (13.2, 21.0)	17.0 (9.5, 24.5)			
6 months	18.0 (11.6, 24.4)	18.4 (10.4, 26.3)			
12 months	20.3 (11.9, 28.7)	16.3 (12.3 20.3)			
Fat intake, kcal percentage			0.68	0.10	0.26
Baseline	35.3 (25.9, 44.7)	35.4 (18.5, 52.2)			
6 months	36.5 (21.2, 51.9)	34.0 (19.6, 48.4)			
12 months	39.9 (20.0, 59.8)	31.8 (24.3, 39.4)			
Carbohydrate intake, kcal percentage^a,c^			0.37	<0.001	0.80
Baseline	50.9 (39.4, 62.5)	47.4 (32.4, 62.5)			
6 months	48.7 (34.5, 63.0)	47.5 (31.2, 63.8)			
12 months	52.0 (35.8, 68.3)	42.0 (34.0, 50.1)			
Total estimated energy intake, kcals^a^			0.64	0.02	0.56
Baseline	1,122.6 (858.1, 1,387.0)	1,086 (657.0, 1,515.9)			
6 months	1,016.6 (664.1, 1,369.1)	983.1 (604.8, 1,361.5)			
12 months	985.8 (595.2, 1,376.5)	792.5 (624.3, 960.7)			

^(1)^Basic nutritional information regarding daily intake of study children. Values are means (95% CIs); *n* = 30, 23, and 21 in the parent mentor group and 30, 23, and 20 in the community health worker group at baseline and at 6 and 12 months, respectively; tsp: teaspoon; kcals: kilocalories.

^(2)^Linear mixed-model analysis on the entire study population; *p* values are for the individual effects of group and time as well as their interaction.

^(3)a^0–6-month mean difference significance at a *p* value < 0.05; ^b^6–12-month mean difference significance at a *p* value < 0.05: (for this table no significant differences were found at 6–12 months); ^c^0–12-month mean difference significance at a *p* value < 0.05.

**Table 5:** Child Comprehensive Feeding Practices Questionnaire (CFPQ) results^(1)^.

Measure^(3)^	Parent mentor group	Community health worker group	*p* ^(2)^		
			Group	Time	Group × time
Child control (allow child control of eating behavior)^a,c^			0.49	<0.001	0.77
Baseline	2.5 (2.3, 2.8)	2.7 (2.4, 3.0)			
6 months	2.3 (2.0, 2.6)	2.2 (1.9, 2.5)			
12 months	2.3 (2.0, 2.6)	2.3 (1.9, 2.6)			
Emotion regulation (parent uses food to regulate emotion)^a,c^			0.34	<0.001	0.62
Baseline	1.5 (1.2, 1.7)	1.7 (1.4, 2.0)			
6 months	1.4 (1.1, 1.7)	1.3 (1.1, 1.5)			
12 months	1.3 (1.1, 1.5)	1.2 (1.0, 1.3)			
Encourage balance and variety in diet^b,c^			0.009	0.001	0.12
Baseline	4.4 (4.2, 4.6)	4.3 (4.0, 4.5)			
6 months	4.5 (4.2, 4.8)	4.3 (4.0, 4.5)			
12 months	4.8 (4.6, 5.0)	4.5 (4.2, 4.7)			
Environment (make healthy foods available in home)^a,b,c^			0.22	<0.001	0.48
Baseline	3.7 (3.3, 4.1)	3.4 (3.0, 3.7)			
6 months	4.4 (4.2, 4.7)	4.1 (3.8, 4.3)			
12 months	4.3 (4.0, 4.5)	3.9 (3.6, 4.2)			
Food as reward for behavior^a,c^			0.17	<0.001	0.39
Baseline	2.4 (2.0, 2.8)	2.6 (2.2, 2.9)			
6 months	1.8 (1.4, 2.1)	2.2 (1.7, 2.6)			
12 months	2.0 (1.6, 2.4)	2.3 (1.8, 2.8)			
Encourage child involvement in meal planning/preparation^a,c^			0.45	<0.001	0.78
Baseline	3.1 (2.8, 3.5)	3.1 (2.7, 3.5)			
6 months	3.6 (3.1, 4.1)	3.5 (3.1, 3.8)			
12 months	3.6 (3.1, 4.1)	3.4 (3.0, 3.9)			
Modeling (parent demonstrates healthy eating)^a,c^			0.31	<0.001	0.53
Baseline	3.8 (3.5, 4.2)	3.7 (3.3, 4.1)			
6 months	4.6 (4.2, 4.9)	4.4 (4.1, 4.7)			
12 months	4.5 (4.2, 4.8)	4.2 (3.8, 4.6)			
Monitoring (parent tracks less healthy foods)			0.82	0.24	0.96
Baseline	3.7 (3.3, 4.0)	3.5 (3.2, 3.9)			
6 months	3.6 (3.1, 4.2)	3.3 (2.9, 3.8)			
12 months	3.8 (3.4, 4.3)	3.6 (3.1, 4.1)			
Pressure (parent pressures child to eat more food at meals)^a,c^			0.63	0.006	0.65
Baseline	2.6 (2.2, 3.0)	2.2 (1.9, 2.5)			
6 months	2.2 (1.8, 2.7)	2.2 (1.7, 2.6)			
12 months	2.2 (1.7, 2.6)	1.9 (1.4, 2.4)			
Restriction for health (parent controls intake to restrict less healthy foods)^a^			0.11	0.05	0.04
Baseline	3.9 (3.5, 4.2)	3.7 (3.4, 4.1)			
6 months	4.4 (4.1, 4.6)	3.8 (3.3, 4.3)			
12 months	4.1 (3.7, 4.4)	4.0 (3.6, 4.4)			
Restriction for weight control (parent controls intake to influence weight)^a,c^			0.13	0.05	0.37
Baseline	2.8 (2.4, 3.2)	3.1 (2.7, 3.5)			
6 months	3.3 (2.9, 3.7)	3.2 (2.8, 3.7)			
12 months	3.3 (2.8, 3.7)	3.2 (2.7, 3.7)			
Teaching about nutrition (explicit instruction to encourage healthy foods)^a,c^			0.37	<0.001	0.21
Baseline	3.9 (3.5, 4.2)	3.6 (3.2, 3.9)			
6 months	4.5 (4.2, 4.8)	4.0 (3.7, 4.4)			
12 months	4.3 (4.0, 4.6)	4.2 (3.8, 4.5)			

^(1)^The CFPQ scales (range: 1–5) with higher scores indicating more agreement with the feeding behavior or higher frequency of the practice. Values are means (95% CIs); *n* = 30, 23, and 21 in the parent mentor group and 30, 23, and 20 in the community health worker group at baseline and at 6 and 12 months, respectively.

^(2)^Linear mixed-model analysis on the entire study population; *p* values are for the individual effects of group and time as well as their interaction.

^(3)a^0–6-month mean difference significance at a *p* value < 0.05; ^b^6–12-month mean difference significance at a *p* value < 0.05; ^c^0–12-month mean difference significance at a *p* value < 0.05.
